# Defucosylated Monoclonal Antibody (H_2_Mab-139-mG_2a_-f) Exerted Antitumor Activities in Mouse Xenograft Models of Breast Cancers against Human Epidermal Growth Factor Receptor 2

**DOI:** 10.3390/cimb45100488

**Published:** 2023-09-23

**Authors:** Hiroyuki Suzuki, Tomokazu Ohishi, Ren Nanamiya, Manabu Kawada, Mika K. Kaneko, Yukinari Kato

**Affiliations:** 1 Department of Molecular Pharmacology, Tohoku University Graduate School of Medicine, 2-1 Seiryo-machi, Aoba-ku, Sendai 980-8575, Japan; ren.nanamiya.p5@dc.tohoku.ac.jp (R.N.); k.mika@med.tohoku.ac.jp (M.K.K.); 2 Department of Antibody Drug Development, Tohoku University Graduate School of Medicine, 2-1 Seiryo-machi, Aoba-ku, Sendai 980-8575, Japan; 3 Institute of Microbial Chemistry (BIKAKEN), Numazu, Microbial Chemistry Research Foundation, 18-24 Miyamoto, Numazu-shi 410-0301, Japan; ohishit@bikaken.or.jp; 4 Institute of Microbial Chemistry (BIKAKEN), Laboratory of Oncology, Microbial Chemistry Research Foundation, 3-14-23 Kamiosaki, Shinagawa-ku, Tokyo 141-0021, Japan; kawadam@bikaken.or.jp

**Keywords:** antibody-dependent cellular cytotoxicity, breast cancer, HER2, monoclonal antibody, antitumor activities, mouse tumor model

## Abstract

The clinically approved human epidermal growth factor receptor 2 (HER2)-targeting monoclonal antibodies (mAbs), trastuzumab, and pertuzumab, target domains IV and II, respectively. Trastuzumab is now the standard treatment for HER2-overexpressed breast and gastric cancers, and trastuzumab in combination with pertuzumab showed clinical benefit. However, there still exist patients who do not respond to the therapy. Furthermore, HER2 mutants that cannot be recognized by pertuzumab were found in tumors. Therefore, novel anti-HER2 mAbs and modalities have been desired. In our previous study, we developed a novel anti-HER2 domain I mAb, H_2_Mab-139 (mouse IgG_1_, kappa). We herein produced a defucosylated mouse IgG_2a_ type of mAb against HER2 (H_2_Mab-139-mG_2a_-f) to enhance antibody-dependent cellular cytotoxicity (ADCC)-mediated antitumor activity. H_2_Mab-139-mG_2a_-f exhibits a high binding affinity in flow cytometry with the dissociation constant (*K*_D_) determined to be 3.9 × 10^−9^ M and 7.7 × 10^−9^ M against HER2-overexpressed Chinese hamster ovary (CHO)-K1 (CHO/HER2) and HER2-positive BT-474 cells, respectively. Moreover, we showed that H_2_Mab-139-mG_2a_-f exerted ADCC and complement-dependent cytotoxicity against CHO/HER2 and BT-474 in vitro and exhibited potent antitumor activities in mouse xenograft models. These results indicated that H_2_Mab-139-mG_2a_-f exerts antitumor effects against HER2-positive human breast cancers and is useful as an antibody treatment for HER2-positive human cancers.

## 1. Introduction

Human epidermal growth factor receptor 2 (HER2) is included in the receptor tyrosine kinase family of human epidermal growth factor receptor (EGFR). The HER activation is controlled by EGF-family ligands under physiological conditions. The formation of multiple combinations of HER homo- and heterodimers is induced by ligand binding, which triggers the activation of the cytoplasmic tyrosine kinase domain. The activation of several downstream signaling pathways, such as the RAS/RAF/MAPK and PI3K/AKT pathways [1], is induced by the autophosphorylation of specific tyrosine residues. HER2 does not have ligands and cannot form ligand-dependent homodimers, unlike EGFR, HER3, and HER4. To activate the downstream signaling, HER2 must either form heterodimers with other HER members and their specific ligands or self-assemble into ligand-independent homodimers when overexpressed. HER2 possesses four extracellular domains (I–IV) [2]. Domain II is known to be essential for the heterodimer formation with other HER members, such as EGFR, HER3, and HER4 in the presence of their ligands, such as EGF [3] and neuregulin 1 (NRG1, a HER3 ligand) [4].

HER2 is overexpressed in approximately 18% of breast cancers and is associated with higher rates of recurrence, poor prognosis, and shorter overall survival [5]. HER2 overexpression is also observed in ~20% of gastric cancers [6]. A monoclonal antibody (mAb) against domain IV of HER2, trastuzumab, exhibited an anti-proliferating effect in vitro and a potent antitumor effect in vivo [7,8]. The addition of trastuzumab to chemotherapy improves objective response rates, progression-free survival, and overall survival in HER2-positive breast cancer patients with metastasis [9]. Trastuzumab has become the standard treatment for HER2-positive breast cancers [10] and HER2-positive gastric cancers [11]. For more than 20 years, trastuzumab has been the most effective therapy for HER2-positive breast cancer [12].

Clinically, the efficacy of trastuzumab involves immunologic engagement [8]. The Fc domain of trastuzumab mediates engagement with Fcγ receptors (FcγRs) on various immune cells. The binding of trastuzumab to FcγR facilitates the phagocytosis of antibody-bound tumor cells, a process known as antibody-dependent cellular phagocytosis (ADCP). The FcγR engagement also activates macrophages, dendritic cells, and neutrophils, that change adaptive immune responses by antigen presentation, cytokine production, and chemotaxis. Moreover, the FcγR engagement activates natural killer (NK) cells, which can result in the lysis of the target tumor cells, termed antibody-dependent cellular cytotoxicity (ADCC) [13]. To improve the FcγRIIIA engagement and ADCC activity, margetuximab was developed by introducing several optimization mutations of trastuzumab [14]. Margetuximab was approved by the U.S. Food and Drug Administration (FDA) and showed significant improvement in progression-free survival in heavily pretreated patients [15,16]. Moreover, the Fc domain of these mAbs can exert complement-dependent cytotoxicity (CDC) [17,18].

Another clinically approved HER2-targeting mAb, pertuzumab, binds to the domain II and prevents NRG1-induced heterodimerization with HER3 and intracellular signaling [19]. The heterodimerization is known to be an important mechanism for resistance to trastuzumab [19]. Therefore, pertuzumab is considered to possess a complementary mechanism to trastuzumab [20]. The first-line treatment combining trastuzumab, pertuzumab, and chemotherapy has been evaluated and demonstrated clinical benefits [21]. The double anti-HER2 blockade has been the standard therapy in the initial management of metastatic HER2-positive breast cancer [9]. However, HER2 (S310F/Y) is the most frequent oncogenic missense mutation which cannot be recognized by pertuzumab [22].

In our previous studies, we established anti-HER2 mAbs including H_2_Mab-139 (IgG_1_, kappa) [23] by the immunization of HER2 ectodomain. Those mAbs have been revealed to recognize the domain I of HER2, and are available for flow cytometry, western blotting, and immunohistochemistry (IHC) [23]. Mouse IgG_1_ cannot bind to mouse FcγRIV which is essential for the activation of effector cells such as macrophage. In contrast, mouse IgG_2a_ or IgG_2b_ can bind to it with high affinity [24]. Furthermore, a core fucose deficiency on the Fc *N*-glycan has been shown to enhance the binding to Fc receptors on effector cells [25]. Therefore, we have demonstrated that class-switched (from IgG_1_ to IgG_2a_) and defucosylated IgG_2a_ mAbs exhibited a superior ability to activate effector cells and exerted potent antitumor effects in several mouse xenograft models [26]. The defucosylated recombinant mAbs can be produced using fucosyltransferase 8 (FUT8)-knockout (KO) Expi-CHO-S cells [27].

In this study, we produced a defucosylated IgG_2a_ type of anti-HER2 mAb (H_2_Mab-139-mG_2a_-f) and evaluated the ability to induce ADCC/CDC in vitro or antitumor efficacy in vivo against HER2-positive and HER2-negative breast cancer cells.

## 2. Materials and Methods

### 2.1. Cell Lines

LN229, BT-474, MDA-MB-468, HEK-293T, and Chinese hamster ovary (CHO)-K1 cell lines were obtained from the American Type Culture Collection (ATCC, Manassas, VA, USA). LN229/HER2 and CHO/HER2 were established as described previously [23]. The HEK-293T/HER2-KO cell line (BINDS-23) was generated by transfecting a CRISPR/Cas9 plasmid that targets HER2 [Thermo Fisher Scientific, Inc. (Thermo), Waltham, MA, USA]. CHO-K1 and CHO/HER2 cell lines were cultured in RPMI-1640 medium [Nacalai Tesque, Inc. (Nacalai), Kyoto, Japan]. LN229, BT-474, BINDS-23, and MDA-MB-468 cell lines were cultured in a DMEM medium (Nacalai). Both media were supplemented with 100 units/mL of penicillin, 100 μg/mL streptomycin, 0.25 μg/mL amphotericin B (Nacalai), and 10% fetal bovine serum (FBS, Thermo, Waltham, MA, USA). All cell lines were cultured at 37 °C in a humidified atmosphere with 5% CO_2_ and 95% air.

### 2.2. Recombinant mAb Production

Anti-HER2 mAb H_2_Mab-139 was established as previously described [23]. To generate H_2_Mab-139-mG_2a_-f, V_H_ cDNA of H_2_Mab-139 and C_H_ of mouse IgG_2a_ were cloned into the pCAG-Ble vector [FUJIFILM Wako Pure Chemical Corporation (Wako), Osaka, Japan]. V_L_ cDNA of H_2_Mab-139 and C_L_ cDNA of mouse kappa light chain were also cloned into the pCAG-Neo vector (Wako). The vectors were transfected into FUT8-knockout ExpiCHO-S (BINDS-09) cells as described previously [28]. H_2_Mab-139-mG_2a_-f was purified using Ab-Capcher (ProteNova Co., Ltd., Kagawa, Japan).

### 2.3. Animal Experiments for ADCC Assay and Mice Xenograft Model

Every animal experiment for ADCC and antitumor activity by H_2_Mab-139-mG_2a_-f was approved by the Institutional Committee for Experiments of the Institute of Microbial Chemistry (Numazu, Japan; approval no. 2022-056, 2023-001, and 2023-018). Mice were monitored and maintained as described previously [28].

### 2.4. Flow Cytometry

CHO-K1, CHO/HER2, LN229, LN229/HER2, BT-474, and MDA-MB-468 were collected using 0.25% trypsin and 1 mM ethylenediamine tetraacetic acid (EDTA) (Nacalai). The cells (1 × 10^5^ cells/sample) were incubated with blocking buffer (control) (0.1% BSA in PBS) or H_2_Mab-139-mG_2a_-f for 30 min at 4 °C. Next, the cells were incubated with Alexa Fluor 488-conjugated anti-mouse IgG (1:2000; Cell Signaling Technology, Inc., Danvers, MA, USA) for 30 min at 4 °C. To confirm the isotype of H_2_Mab-139-mG_2a_-f, the mAb-treated LN229/HER2 cells were incubated with fluorescein-conjugated anti-mouse heavy chains (IgG_1_ and IgG_2a_; SouthernBiotech, Birmingham, AL, USA) or Alexa Fluor 488-conjugated anti-mouse immunoglobulins for 30 min at 4 °C. SA3800 Cell Analyzer (Sony Corp., Tokyo, Japan) was used to collect the fluorescence data, which were analyzed using FlowJo [BD Biosciences (BD), Franklin Lakes, NJ, USA].

### 2.5. Determination of Dissociation Constant (K_D_) via Flow Cytometry

CHO/HER2 and BT-474 were suspended in 100 μL of serially diluted H_2_Mab-139-mG_2a_-f (600 pg/mL–10 μg/mL). After the washing step, those cells were reacted with Alexa Fluor 488-conjugated anti-mouse IgG at a ratio of 1:200 (*n* = 3). Fluorescence data were detected using the SA3800 Cell Analyzer. The apparent dissociation constant (*K*_D_) was calculated using GraphPad Prism 8 (GraphPad Software, Inc., La Jolla, CA, USA) as described previously [26].

### 2.6. Western Blot Analysis

The cell lysates (10 μg) were treated with sodium dodecyl sulfate (SDS) sample buffer (Nacalai) for 5 min at 95 °C. The proteins were electrophoresed using 5–20% polyacrylamide precast gels for electrophoresis (Wako) and transferred to polyvinylidene difluoride (PVDF) membranes (Merck KGaA, Darmstadt, Germany). The membranes were blocked using 4% skim milk (Nacalai) in PBS with 0.05% Tween 20 and were treated with 1 μg/mL of H_2_Mab-139-mG_2a_-f or 1 μg/mL of an anti-isocitrate dehydrogenase 1 (IDH1) mAb (RcMab-1). The membranes were then incubated with anti-mouse immunoglobulins conjugated with peroxidase [diluted 1:1000; Agilent Technologies, Inc. (Agilent), Santa Clara, CA, USA, for H_2_Mab-139-mG_2a_-f], or with anti-rat immunoglobulins conjugated with peroxidase (diluted 1:1000; Agilent, for RcMab-1). Finally, the signals were detected using a chemiluminescence reagent, ImmunoStar LD (Wako) using a Sayaca-Imager (DRC Co., Ltd., Tokyo, Japan).

### 2.7. IHC Analysis

A paraffin-embedded breast cancer tissue microarray (T8235721-5, BioChain Institute Inc., Eureka Drive Newark, CA, USA) was autoclaved for 20 min using Envision FLEX TARGET RETRIEVAL SOLUTION High pH. We used SuperBlock T20 (Thermo) for blocking to inhibit the non-specific binding of mAbs to sections. The sections were treated with 10 μg/mL of H_2_Mab-139-mG_2a_-f for 1 h at room temperature and then incubated with the EnVision+ Kit for mouse (Agilent) for 30 min. The chromogenic reaction and counterstaining were performed using 3,3′-diaminobenzidine tetrahydrochloride (DAB; Agilent) and hematoxylin (Wako), respectively.

### 2.8. ADCC

The splenocytes were obtained from female BALB/c nude mice (five-week-old, Charles River Laboratories, Inc., Tokyo, Japan) as described previously [28], and were used as effector cells. The ADCC by H_2_Mab-139-mG_2a_-f was assayed as follows. CHO-K1, CHO/HER2, BT-474, and MDA-MB-468 were labeled with 10 μg/mL Calcein AM (Thermo). The Calcein AM-labeled target cells (2 × 10^4^ cells) were mixed with the effector cells (effector/target cells ratio, 50), 100 μg/mL of H_2_Mab-139-mG_2a_-f or control mouse IgG_2a_ (mIgG_2a_, Sigma-Aldrich, St. Louis, MO, USA). We incubated them for 4.5 h at 37 °C, and measured the Calcein release into the medium using a microplate reader (Power Scan HT; BioTek Instruments, Inc., Winooski, VT, USA).

We determined the cytotoxicity (% lysis) as follows:

% lysis = (E − S)/(M − S) × 100

“E” indicates the fluorescence in cultures of both effector and target cells,
“S” indicates the spontaneous fluorescence of only target cells,
“M” indicates the maximum fluorescence following the treatment with a lysis buffer [10 mM Tris-HCl (pH 7.4), 10 mM EDTA, and 0.5% Triton X-100].

### 2.9. CDC

We plated the Calcein AM-labeled target cells (CHO-K1, CHO/HER2, BT-474, and MDA-MB-468) and mixed those cells with rabbit complement (final dilution 1:10, Low-Tox-M Rabbit Complement; Cedarlane Laboratories, Hornby, ON, Canada) and 100 μg/mL of control mIgG_2a_ or H_2_Mab-139-mG_2a_-f. We incubated them for 4.5 h at 37 °C, and measured the Calcein release into the medium, as described above.

### 2.10. Antitumor Activity of H_2_Mab-139-mG_2a_-f in Xenografts of CHO-K1, CHO/HER2, BT-474, and MDA-MB-468 Cells

We resuspended CHO/HER2 (5 × 10^6^ cells) in DMEM and mixed them with BD Matrigel Matrix Growth Factor Reduced (BD). We injected them subcutaneously into the left flank of BALB/c nude mice (female, 5 weeks old, Charles River Laboratories, Inc.). On day 8 post-inoculation, 100 μg of H_2_Mab-139-mG_2a_-f (*n* = 8) or control mouse IgG (mIgG, Wako) (*n* = 8) in 100 μL PBS were intraperitoneally injected. On days 14 and 22, additional antibody injections were performed. The tumor volume was measured on days 8, 12, 14, 19, 22, and 26 after the inoculation of cells.

We injected BT-474 and MDA-MB-468 (5 × 10^6^ cells) subcutaneously into the left flank of BALB/c nude mice, as indicated above. On day 7 post-inoculation, 100 μg of H_2_Mab-139-mG_2a_-f (*n* = 8) or control mIgG (*n* = 8) in 100 μL PBS was intraperitoneally injected. On days 14 and 21, additional antibody injections were performed. The tumor volume was measured on days 7, 10, 14, 16, 21, 24, and 28 after the inoculation of cells.

### 2.11. Statistical Analyses

All data are shown as mean ± standard error of the mean (SEM). Welch’s *t*-test was used for the statistical analyses in ADCC, CDC, and tumor weight. ANOVA with Sidak’s post hoc test was used in tumor volume and mouse weight. GraphPad Prism 8 (GraphPad Software, Inc.) was utilized for the calculations. A *p* < 0.05 was considered to indicate a statistically significant difference.

## 3. Results

### 3.1. Detection of HER2 Using H_2_Mab-139-mG_2a_-f by Flow Cytometry

**Figure 1:**
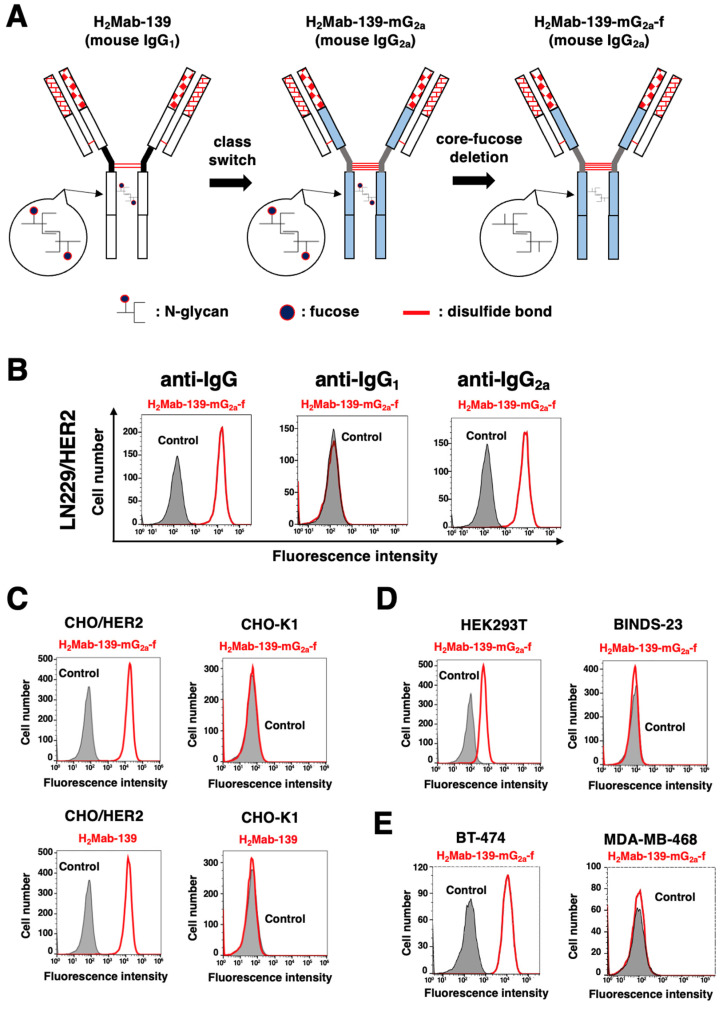
Flow cytometry using H_2_Mab-139-mG_2a_-f. (**A**) A core-fucose-deficient mouse IgG_2a_ mAb, H_2_Mab-139-mG_2a_-f was produced from H_2_Mab-139 (mouse IgG_1_). (**B**) LN229/HER2 cells were treated with 1 µg/mL of H_2_Mab-139-mG_2a_-f (red) or buffer control (filled gray), followed by Alexa Fluor 488-conjugated anti-mouse IgG or Fluorescein-conjugated anti-mouse heavy chains (IgG_1_ and IgG_2a_). (**C**) CHO-K1 and CHO/HER2 cells were treated with 10 µg/mL of H_2_Mab-139-mG_2a_-f (red), H_2_Mab-139 (red), or buffer control (filled gray), followed by Alexa Fluor 488-conjugated anti-mouse IgG. (**D**) HEK293T and HER2-KO HEK293T (BINDS-23) cells were treated with 10 µg/mL of H_2_Mab-139-mG_2a_-f (red) or buffer control (filled gray), followed by Alexa Fluor 488-conjugated anti-mouse IgG. (**E**) Breast cancer cell lines, BT-474 and MDA-MB-468 cells were treated with 10 µg/mL of H_2_Mab-139-mG_2a_-f (red) or buffer control (filled gray), followed by Alexa Fluor 488-conjugated anti-mouse IgG.

We previously established an anti-HER2 mAb (H_2_Mab-139, IgG_1_, kappa) by the immunization of the HER2 ectodomain produced by glioblastoma LN229 cells [23]. H_2_Mab-139 was advantageous for western blotting, flow cytometry, and IHC [23]. Here, we engineered a class-switched and defucosylated H_2_Mab-139 (H_2_Mab-139-mG_2a_-f) by fusing the V*_H_* chains of H_2_Mab-139 with the C*_H_* chains of mouse IgG_2a_ (Figure 1A). We confirmed that H_2_Mab-139-mG_2a_-f was selectively recognized by anti-IgG_2a_, but not anti-IgG_1_ secondary antibodies in flow cytometry (Figure 1B). H_2_Mab-139-mG_2a_-f detected CHO/HER2 cells, but not parental CHO-K1 cells (Figure 1C). The reactivity did not change compared to the original H_2_Mab-139 (Figure 1C). Furthermore, H_2_Mab-139-mG_2a_-f reacted with HEK-293T cells, but not with HER2-KO HEK293T (BINDS-23) cells (Figure 1D). We next investigated the reactivity of H_2_Mab-139-mG_2a_-f against breast cancer cell lines. As shown in Figure 1E, H_2_Mab-139-mG_2a_-f reacted HER2-positive breast cancer cell line, BT-474, but not the triple-negative breast cancer (TNBC) cell line, MDA-MB-468.

A kinetic analysis of the interactions of H_2_Mab-139-mG_2a_-f with CHO/HER2 and BT-474 was performed by flow cytometry. The apparent *K*_D_ for the interaction of H_2_Mab-139-mG_2a_-f with CHO/HER2 and BT-474 were 3.9 × 10^−9^ M and 7.7 × 10^−9^ M, respectively. These results suggest that H_2_Mab-139-mG_2a_-f demonstrates a high affinity for HER2-expressing cells.

### 3.2. Detection of HER2 Using H_2_Mab-139-mG_2a_-f in Western Blot and IHC Analyses

**Figure 2:**
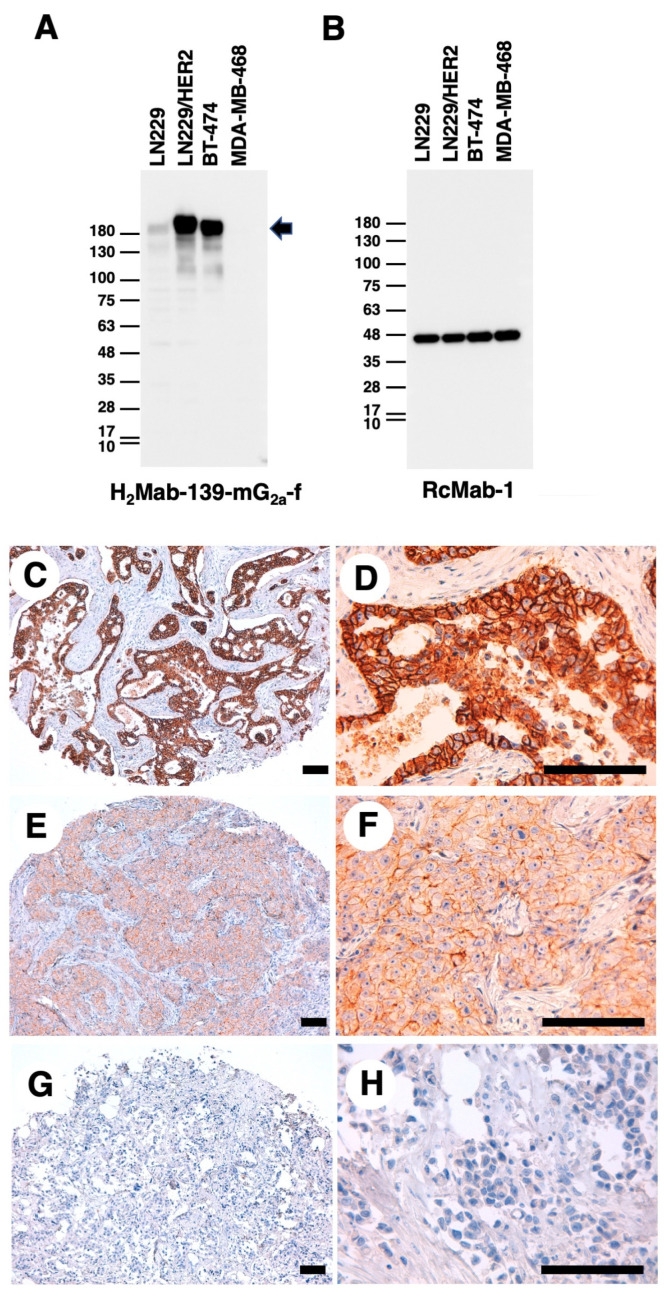
Western blot and IHC analyses using H_2_Mab-139-mG_2a_-f. (**A**,**B**) Western blotting using H_2_Mab-139-mG_2a_-f. The cell lysates of LN229, LN229/HER2, BT-474, and MDA-MB-468 were electrophoresed and transferred onto polyvinylidene fluoride membranes. The membranes were incubated with 1 µg/mL of H_2_Mab-139-mG_2a_-f (**A**) or 1 µg/mL of RcMab-1 (an anti-IDH mAb) (**B**). The arrow indicates the predicted size of HER2 (~180 kDa). An arrow indicates the band of HER2. (**C**–**H**) IHC analysis of a breast cancer tissue array using H_2_Mab-139-mG_2a_-f. Scale bar = 100 μm.

We next performed western blot analysis using H_2_Mab-139-mG_2a_-f. As shown in Figure 2A, H_2_Mab-139-mG_2a_-f strongly detected HER2 as more than 180-kDa bands in LN229/HER2 and BT-474 cells. H_2_Mab-139-mG_2a_-f faintly detected endogenous HER2 in LN229 cells, but not MDA-MB-468 cells. The expression of IDH1 detected by RcMab-1 was used as an internal control (Figure 2B). These results indicated that H_2_Mab-139-mG_2a_-f could detect exogenous and endogenous HER2 in western blot analysis.

Next, IHC analyses against the formalin-fixed paraffin-embedded (FFPE) sections of breast cancer tissue were performed using H_2_Mab-139-mG_2a_-f. As shown in Figure 2C–H, H_2_Mab-139-mG_2a_-f could distinguish HER2-strong positive (Figure 2C,D), moderate (Figure 2E,F), and negative (Figure 2G,H) breast cancers. The HER2-positive staining was mainly observed on the plasma membrane. We summarized the results of HER2 expression in breast cancer tissue array in Supplementary Table S1. H_2_Mab-139-mG_2a_-f stained 10 out of 63 cases (16%) of breast cancers. These results indicated that H_2_Mab-139-mG_2a_-f is also available for IHC analysis of FFPE tumor sections.

### 3.3. ADCC and CDC by H_2_Mab-139-mG_2a_-f against CHO/HER2 Cells

**Figure 3:**
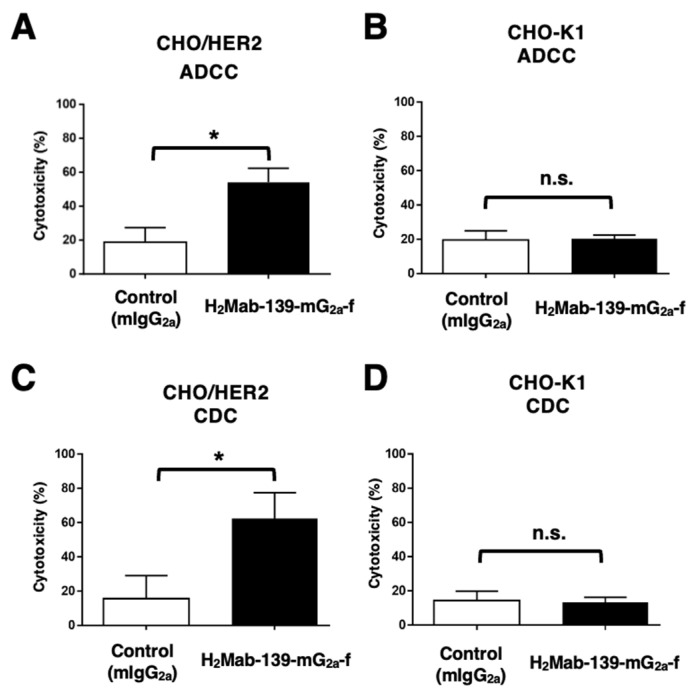
H_2_Mab-139-mG_2a_-f-mediated ADCC and CDC activities in CHO/HER2 and CHO-K1 cells. (**A**,**B**) ADCC induced by H_2_Mab-139-mG_2a_-f or control mouse IgG_2a_ (mIgG_2a_) against CHO/HER2 (**A**) and CHO-K1 (**B**) cells. (**C**,**D**) CDC induced by H_2_Mab-139-mG_2a_-f or control mIgG_2a_ against CHO/HER2 (**C**) and CHO-K1 (**D**) cells. Values are shown as mean ± SEM. Asterisks indicate statistical significance (* *p* < 0.05; Welch’s *t*-test). n.s., not significant.

We next investigated whether H_2_Mab-139-mG_2a_-f could exert ADCC against CHO/HER2 cells. H_2_Mab-139-mG_2a_-f showed ADCC (54.1% cytotoxicity) against CHO/HER2 cells more effectively than the control mouse IgG_2a_ (18.8% cytotoxicity; *p* < 0.05) (Figure 3A). No difference was observed between H_2_Mab-139-mG_2a_-f and control mIgG_2a_ about ADCC against CHO-K1 (Figure 3B).

We then examined whether H_2_Mab-139-mG_2a_-f could exhibit CDC against CHO/HER2 cells. As shown in Figure 3C, H_2_Mab-139-mG_2a_-f elicited a higher degree of CDC (62.5% cytotoxicity) in CHO/HER2 cells compared with that elicited by control mIgG_2a_ (15.6% cytotoxicity; *p* < 0.05). There was no difference between H_2_Mab-139-mG_2a_-f and control mIgG_2a_ in CDC for CHO-K1 (Figure 3D). These results showed that H_2_Mab-139-mG_2a_-f exerted significantly high levels of ADCC/CDC against CHO/HER2 cells.

### 3.4. Antitumor Effects of H_2_Mab-139-mG_2a_-f in the Mouse Xenografts of CHO/HER2 Cells

**Figure 4:**
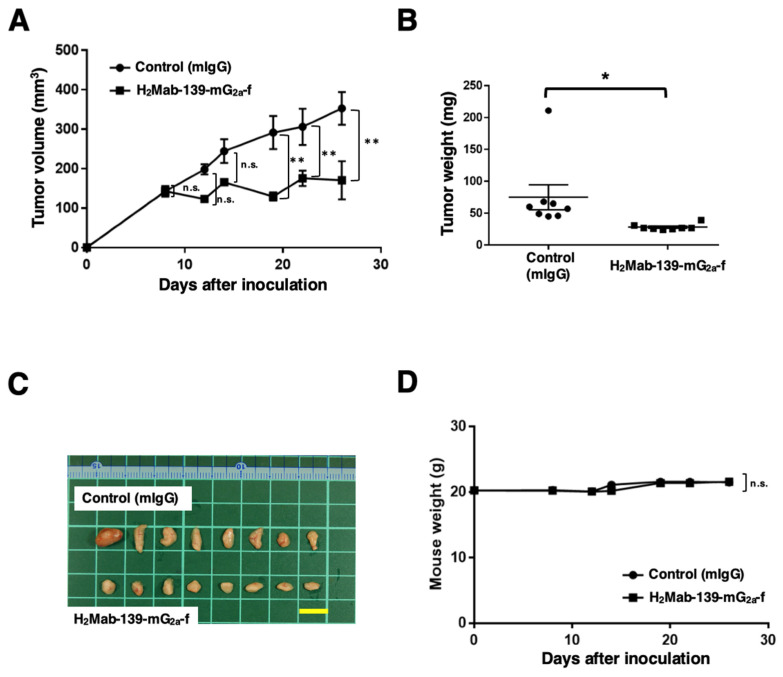
Antitumor activity of H_2_Mab-139-mG_2a_-f against CHO/HER2 xenograft. (**A**) CHO/HER2 cells were subcutaneously injected into BALB/c nude mice (day 0). On day 8, 100 μg of H_2_Mab-139-mG_2a_-f or control normal mouse IgG (mIgG) was injected intraperitoneally into mice. Additional antibodies were injected on days 14 and 22. We measured the tumor volume on days 8, 12, 14, 19, 22, and 26. Values are presented as the mean ± SEM. ** *p* < 0.01 (ANOVA and Sidak’s multiple comparisons test). (**B**) Tumor weight of CHO/HER2 xenograft tumors on day 28. Values are presented as the mean ± SEM. * *p* < 0.05 (Welch’s *t*-test). (**C**) The CHO/HER2 xenograft tumors on day 28 (scale bar, 1 cm). (**D**) Body weight in mIgG and H_2_Mab-139-mG_2a_-f treated mice. n.s., not significant.

Following the inoculation of CHO/HER2, we injected H_2_Mab-139-mG_2a_-f and control mIgG intraperitoneally into CHO/HER2 xenograft tumor-bearing mice on days 8, 14, and 22. On days 8, 12, 14, 19, 22, and 26 after the tumor inoculation, we measured the tumor volume. The H_2_Mab-139-mG_2a_-f administration reduced the tumor volume on days 19 (*p* < 0.01), 22 (*p* < 0.01), and 26 (*p* < 0.01) compared with that of mIgG (Figure 4A). The H_2_Mab-139-mG_2a_-f administration led to a 52% reduction of the tumor volume compared with that of the control mIgG on day 26 post-injection.

The weight of CHO/HER2 tumors treated with H_2_Mab-139-mG_2a_-f was significantly lower than that treated with mIgG (66% reduction; *p* < 0.05; Figure 4B). CHO/HER2 tumors that were resected from mice on day 26 are demonstrated in Figure 4C.

The body weight loss and skin disorders were not detected in CHO/HER2 tumor-bearing mice (Figure 4D). The mice on day 26 were shown in Supplementary Figure S1.

### 3.5. ADCC and CDC by H_2_Mab-139-mG_2a_-f against BT-474 and MDA-MB-468 Cells

**Figure 5:**
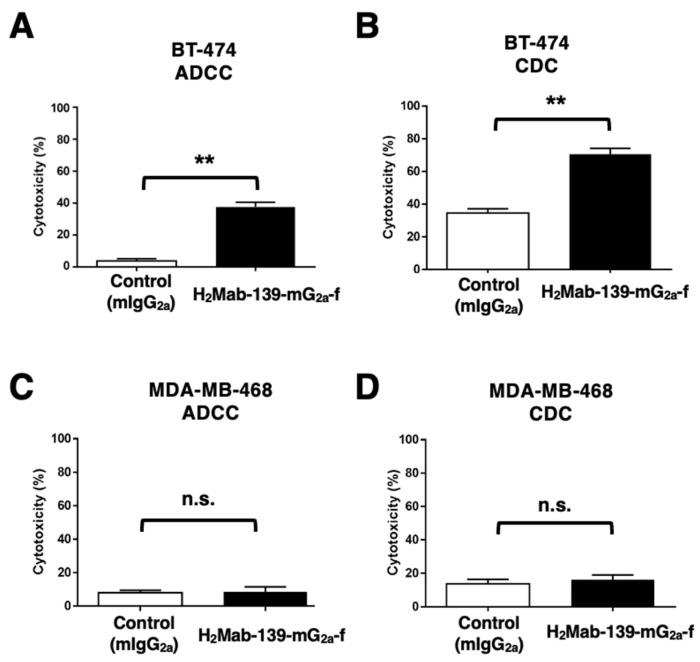
H_2_Mab-139-mG_2a_-f-mediated ADCC and CDC activities in BT-474 (HER2-positive) and MDA-MB-468 (TNBC) cells. (**A**,**C**) ADCC induced by H_2_Mab-139-mG_2a_-f or control mIgG_2a_ against BT-474 (**A**) and MDA-MB-468 (**C**) cells. (**B**,**D**) CDC induced by H_2_Mab-139-mG_2a_-f or control mIgG_2a_ against BT-474 (**B**) and MDA-MB-468 (**D**) cells. Values are shown as mean ± SEM. Asterisks indicate statistical significance (** *p* < 0.01; Welch’s *t*-test). n.s., not significant.

It was investigated whether H_2_Mab-139-mG_2a_-f was capable of mediating ADCC against BT-474 and MDA-MB-468 cells. As revealed in Figure 5A, H_2_Mab-139-mG_2a_-f showed ADCC (37.6% cytotoxicity) against BT-474 cells more potently than did the control mIgG_2a_ (3.8% cytotoxicity; *p* < 0.01). We next investigated whether H_2_Mab-139-mG_2a_-f exhibited CDC against BT-474 cells. H_2_Mab-139-mG_2a_-f showed a significantly high CDC (70.6% cytotoxicity) in BT-474 cells compared with that induced by control mIgG_2a_ (34.6% cytotoxicity; *p* < 0.01) (Figure 5B). However, there was no difference between H_2_Mab-139-mG_2a_-f and control mIgG_2a_ in ADCC (Figure 5C) and CDC (Figure 5D) against MDA-MB-468. These results demonstrated that H_2_Mab-139-mG_2a_-f exhibited higher levels of ADCC and CDC against HER2-positive BT-474 cells.

### 3.6. Antitumor Effects of H_2_Mab-139-mG_2a_-f in BT-474 and MDA-MB-468 Xenografts

**Figure 6:**
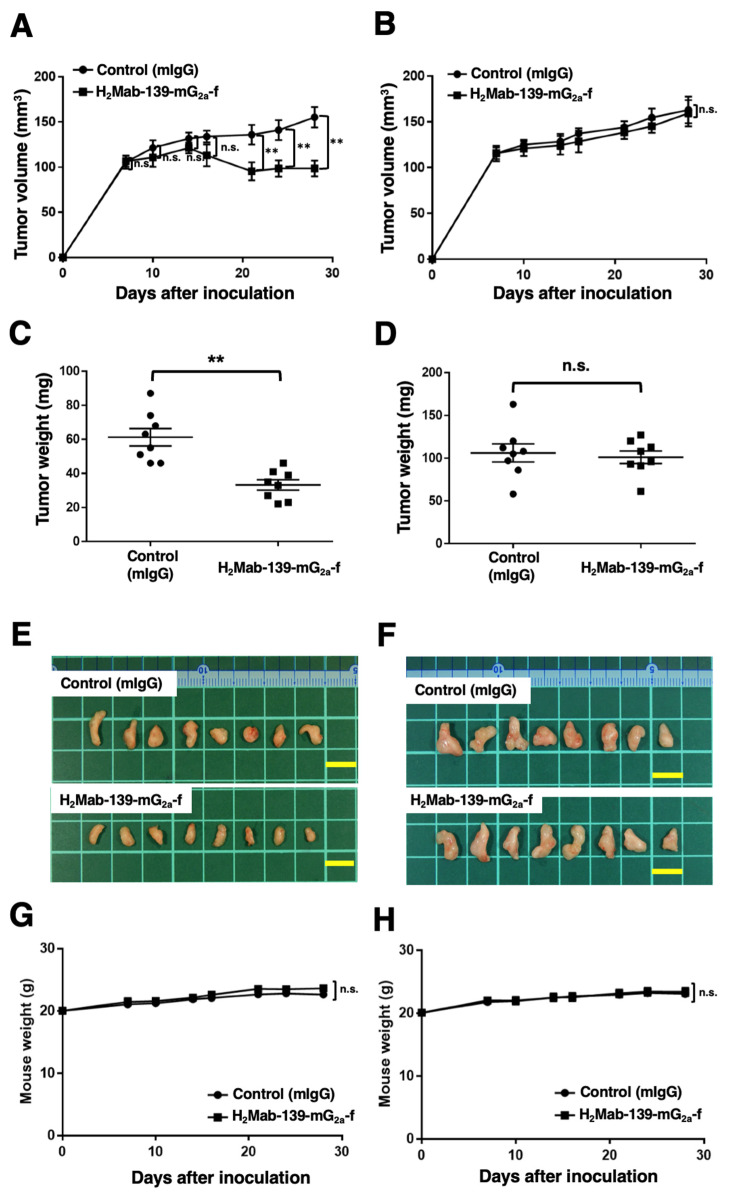
Antitumor activity of H_2_Mab-139-mG_2a_-f against BT-474 and MDA-MB-468 xenografts. (**A**,**B**) BT-474 (**A**) and MDA-MB-468 (**B**) cells were injected into BALB/c nude mice (day 0). On day 7, 100 μg of H_2_Mab-139-mG_2a_-f or control normal mouse IgG (mIgG) were injected into mice. On days 14 and 21, additional antibodies were injected. On days 7, 10, 14, 16, 21, 24, and 28, the tumor volume was measured. Values are presented as the mean ± SEM. ** *p* < 0.01 (ANOVA and Sidak’s multiple comparisons test). (**C**,**D**) Tumor weight of BT-474 (**C**) and MDA-MB-468 (**D**) xenograft tumors on day 28. Values are presented as the mean ± SEM. ** *p* < 0.01 (Welch’s *t*-test). (**E**,**F**) The BT-474 (**E**) and MDA-MB-468 (**F**) xenograft tumors on day 28. Scale bar, 1 cm. (**G**,**H**) The body weight of BT-474 (**G**) and MDA-MB-468 (**H**) xenograft-bearing mice treated with mIgG and H_2_Mab-139-mG_2a_-f. n.s., not significant.

In the BT-474 xenograft models, we injected H_2_Mab-139-mG_2a_-f and control mIgG intraperitoneally on days 7, 14, and 21 after BT-474 inoculation. We measured the tumor volume on days 7, 10, 14, 16, 21, 24, and 28 following the inoculation. The H_2_Mab-139-mG_2a_-f administration led to a significant reduction in BT-474 xenograft on days 21 (*p* < 0.01), 24 (*p* < 0.01), and 28 (*p* < 0.01) compared with that of the control mIgG (Figure 6A). The H_2_Mab-139-mG_2a_-f administration resulted in a 36% reduction of tumor volume compared with that of the control mIgG on day 28.

Tumors from the H_2_Mab-139-mG_2a_-f-treated mice weighed significantly less than those from the control mIgG-treated mice (45% reduction; *p* < 0.01, Figure 6C). We resected tumors from mice on day 28 (Figure 6E).

In the MDA-MB-468 xenograft models, H_2_Mab-139-mG_2a_-f and control mIgG were injected into mice on days 7, 14, and 21 intraperitoneally after the inoculation of MDA-MB-468 cells. The tumor volume was measured on days 7, 10, 14, 16, 21, 24, and 28. No difference was observed between H_2_Mab-139-mG_2a_-f and control mIgG about MDA-MB-468 xenograft volume (Figure 6B) and weight (Figure 6D). MDA-MB-468 tumors that were resected from mice on day 28 are depicted in Figure 6F.

The body weight loss was not detected in both BT-474 and MDA-MB-468 xenograft-bearing mice (Figure 6G,H). The mice on day 28 about BT-474 and MDA-MB-468 xenograft were demonstrated in Supplementary Figure S2.

## 4. Discussion

Trastuzumab is clinically administered for patients with HER2-overexpressing metastatic breast cancers, which are defined by strong and complete IHC membranous staining of more than 10% of cells (IHC 3+) and/or in situ hybridization (ISH)-amplified. Based on clinical studies, the 5th European School of Oncology and the European Society of Medical Oncology guidelines for advanced breast cancer (ABC 5) and the National Comprehensive Cancer Network guidelines consider trastuzumab (anti-HER2 domain IV mAb), pertuzumab (anti-HER2 domain II mAb), and docetaxel as the standard of care for first-line treatment of HER2-positive metastatic breast cancer [29]. However, most deaths in the study were due to breast cancer [21]. Therefore, better treatments including novel combination therapies and novel modalities are still needed. In this study, we evaluated a novel anti-HER2 domain I mAb, H_2_Mab-139-mG_2a_-f, and showed the ADCC activity in vitro (Figure 3 and Figure 5) and antitumor effect in vivo (Figure 4 and Figure 6). Therefore, H_2_Mab-139-mG_2a_-f could be an antibody treatment regimen for HER2-positive breast cancer.

The structures of the HER2–HER3–NRG1β complex, revealed by cryo-EM, exhibit a dynamic dimer interface. In the complex, the NRG1β-bound HER3 dimerization arm remains unresolved due to the lack of a ligand-induced conformational change in the apo HER2 monomer, which is essential for the formation of the HER3 dimerization arm-binding pocket [22]. In contrast, the most frequent oncogenic HER2 mutation (S310F/Y) was found primarily in cancers without HER2 overexpression. The HER2 S310 is localized in the dimerization arm-binding pocket of domain II [30]. The structures of HER2 (S310F)–HER3–NRG1β complex exhibited stabilizing interactions with the HER3 dimerization arm and compensate for the inability of HER2 to undergo a needed conformational change [22]. Furthermore, HER2–HER3 and HER2 (S310F)–HER3 retain the ability to bind to trastuzumab, but the mutant complex does not bind to pertuzumab [22]. These results suggest that pertuzumab is less effective at targeting cancers driven by HER2 (S310F), and different epitope-possessing anti-HER2 mAbs including H_2_Mab-139 could be required for the combination therapy with trastuzumab.

Trastuzumab-based antibody-drug conjugates (ADCs) including trastuzumab-deruxtecan (T-DXd) have been evaluated. These ADCs rely on the direct cytotoxicity of the released DXd (a DNA topoisomerase I inhibitor) following endocytosis of the HER2-bound mAbs-drug conjugate [31]. T-DXd initially exhibited beneficial outcomes in patients with metastatic breast cancer, who had undergone multiple anti-HER2-targeting treatments [32]. Currently, various clinical trials are evaluating the efficacy of T-DXd. Based on studies, T-DXd has been approved in not only HER2-positive breast cancer [33,34,35], but also HER2-mutant lung cancer [34] and HER2-low (IHC 1+ or IHC 2+/ISH-non-amplified) advanced breast cancer [33].

Given that approximately half of all breast cancers are classifiable as HER2-low [36], a greater number of patients may benefit from T-DXd therapy. These results have had a significant impact on the field of breast oncology, particularly in the future clinical diagnostics of HER2-low breast cancer. As a result, future treatment algorithms for both hormone receptor-positive and TNBC are anticipated to change [37]. There are several challenges in elucidating the biological roles and pathological significance of HER2-low [38]. Since our H_2_Mab-139 is applicable for IHC (Figure 2), it would be valuable to compare its reactivity with approved anti-HER2 diagnostic mAbs such as HercepTest^TM^ and PATHWAY^®^.

We achieved increased ADCC activity of H_2_Mab-139-mG_2a_-f through class switching and a core fucose deficiency on the *N*-glycan in the Fc region, which promotes the binding of Fc to FcγRIIIa on effector cells [25]. This technique is also applied to mogamulizumab (Poteligeo), a defucosylated humanized mAb targeting CCR4 [39]. In contrast, margetuximab is derived from trastuzumab and shares the same epitope with HER2. Five amino acid substitutions in the Fc domain of margetuximab (human IgG_1_) achieve increased binding to FcγRIIIa and reduced binding to an inhibitory FcγR, FcγRIIb, when compared to trastuzumab [9]. We are going to apply the strategy to potentiate the ADCC activity when we generate the humanized H_2_Mab-139 mAb.

We previously produced a bispecific Ab against EGFR and HER2 from our established anti-EGFR mAb (EMab-134) and an anti-HER2 mAb (H_2_Mab-77) [40]. The bispecific Ab possesses the tetravalent structure by fusing the single chain Fv of H_2_Mab-77 at the light chains of EMab-134 and showed the antitumor effect in the mouse xenograft model [40]. Since we can produce the different types of bispecific Abs and have various clones of anti-HER2 mAbs including H_2_Mab-139 (see Supplementary Materials), we will investigate the activity in future studies.

Previously, we established H_2_Mab-139 using cancer cell-produced HER2 ectodomain as an immunogen. This methodology is essential for the development of cancer-specific mAbs (CasMabs). We have developed CasMabs that target podoplanin (PDPN) [41], which recognize the aberrant glycosylation patterns typical of cancer cells [42]. Anti-PDPN-CasMabs are currently applied to CAR-T therapy in preclinical models [43,44]. For the development of anti-HER2 CasMab, we need to perform further screening of our already established anti-HER2 mAbs (more than 200 clones), comparing their reactivity against normal cells [45,46]. Anti-HER2 CasMabs could be employed in designing modalities including ADCs and CAR-T.

## Data Availability

The data presented in this study are available in the article and Supplementary Material.

## Supplementary Materials

The following supporting information can be downloaded at: https://www.mdpi.com/article/10.3390/cimb45100488/s1, Figure S1: Body appearance in CHO/HER2 xenografts-implanted mice treated with mIgG and H_2_Mab-139-mG_2a_-f on day 26. Figure S2: Body appearance in (**A**) BT-474 and (**B**) MDA-MB-468 xenografts-implanted mice treated with mIgG and H_2_Mab-139-mG_2a_-f on day 28. Table S1: IHC analysis using H_2_Mab-139-mG_2a_-f against breast cancer tissue array.
