# Supplementary material for: Defucosylated Monoclonal Antibody (H2Mab-139-mG2a-f) Exerted Antitumor Activities in Mouse Xenograft Models of Breast Cancers against Human Epidermal Growth Factor Receptor 2
Source: Curr Issues Mol Biol. 2023 Sep 23;45(10):7734–48. doi: 10.3390/cimb45100488 (PMC10605610; doi:10.3390/cimb45100488)
Supplement: Supplementary file 1 [file cimb-45-00488-s001.zip › Table S1.pdf]

Table S1. Immunohistochemical analysis using H_2Mab-139-mG_{2a}-f against breast cancer tissue array

No.	Age	Pathological diagnosis	Differentiation	TNM	H_2Mab-139-mG_{2a}-f
1	44	Invasive ductal carcinoma	Moderately	T2N2M1	−
2	58	Medullary carcinoma	Moderately	T2N2M1	−
3	40	Invasive ductal carcinoma	Moderately	T2N1M0	++
4	52	Invasive ductal carcinoma	Moderately	T2N2M1	−
5	60	Invasive ductal carcinoma	Moderately	T2N1M1	−
6	57	Invasive ductal carcinoma	Moderately	T2N0M0	−
7	48	Invasive ductal carcinoma	Moderately	T2N0M0	+++
8	66	Invasive ductal carcinoma	Moderately	T2N0M0	−
9	58	Adenocarcinoma	Moderately	T2N2M1	−
10	63	Invasive ductal carcinoma	Moderately	T2N0M0	−
11	32	Invasive ductal carcinoma	Moderately	T2N0M0	−
12	59	Invasive lobular carcinoma	Well	T2N2M0	−
13	44	Invasive lobular carcinoma	Well	T2N2M0	−
14	60	Invasive lobular carcinoma	Moderately	T2N1M0	−
15	44	Invasive ductal carcinoma	Moderately	T2N2M0	+++
16	82	Invasive ductal carcinoma	Moderately	T2N1M1	−
17	58	Adenocarcinoma	Moderately	T2N1M1	−
18	57	Invasive ductal carcinoma	Poorly	T3N3M0	−
19	41	Invasive ductal carcinoma	Moderately	T2N1M0	−
20	44	Invasive ductal carcinoma	Moderately	T2N2M0	−
21	78	Invasive ductal carcinoma	Moderately	T2N1M0	−
22	60	Invasive ductal carcinoma	Moderately	T2N0M0	+
23	/	Invasive ductal carcinoma	Moderately	T2N1M1	++
24	46	Invasive ductal carcinoma	Moderately	T2N3M1	−
25	41	Invasive ductal carcinoma	Moderately	T2N2M0	−
26	59	Invasive ductal carcinoma	Poorly	T2N0M0	−
27	45	Invasive ductal carcinoma	Poorly	T2N0M0	−
28	43	Invasive ductal carcinoma	N/A	T2N1M1	−
29	26	Fibroadenoma	N/A	T1N0M0	−
30	40	Invasive ductal carcinoma	N/A	T1N0M0	−
31	38	Fibroadenoma	N/A	T2N0M0	−
32	51	Invasive ductal carcinoma	Moderately	T2N2M0	−

33	45	Invasive ductal carcinoma	Poorly	T2N0M0	++
34	45	Invasive ductal carcinoma	Poorly	T2N1M0	+++
35	47	Invasive ductal carcinoma	Moderately	T2N1M0	−
36	55	Invasive ductal carcinoma	Moderately	T2N3M1	+
37	58	Invasive ductal carcinoma	Moderately	T3N3M0	−
38	47	Invasive ductal carcinoma	Moderately	T2N0M0	−
39	38	Invasive ductal carcinoma	Poorly	T2N0M0	−
40	40	Invasive ductal carcinoma	Poorly	T2N0M0	−
41	57	Invasive ductal carcinoma	Poorly	T2N0M0	−
42	42	Invasive ductal carcinoma	Moderately	T2N0M0	+++
43	60	Invasive ductal carcinoma	Moderately	T2N0M0	−
44	58	Invasive ductal carcinoma	Moderately	T2N0M0	−
45	41	Invasive ductal carcinoma	Moderately	T2N0M0	−
46	50	Invasive ductal carcinoma	Moderately	T2N0M0	−
47	60	Invasive ductal carcinoma	Moderately	T2N2M1	−
48	53	Invasive ductal carcinoma	Moderately	T2N0M0	−
49	65	Invasive ductal carcinoma	Moderately	T2N0M0	−
50	43	Invasive ductal carcinoma	Moderately	T2N0M0	−
51	57	Invasive ductal carcinoma	Moderately	T2N0M0	+++
52	37	Invasive ductal carcinoma	Moderately	T2N0M0	−
53	50	Invasive ductal carcinoma	Moderately	T2N3M0	−
54	48	Invasive ductal carcinoma	Poorly	T2N1M0	−
55	50	Invasive ductal carcinoma	Moderately	T2N0M0	−
56	53	Invasive ductal carcinoma	Moderately	T2N0M0	−
57	49	Invasive ductal carcinoma	Moderately	T2N0M0	−
58	65	Invasive ductal carcinoma	Moderately	T2N1M0	−
59	43	Invasive ductal carcinoma	Moderately	T2N0M0	−
60	58	Invasive ductal carcinoma	Moderately	T2N0M0	−
61	48	Invasive ductal carcinoma	Moderately	T2N0M0	−
62	/	Invasive ductal carcinoma	Moderately	TxNxMx	−
63	/	Invasive ductal carcinoma	Moderately	TxNxMx	−

-, No stain; +, Weak intensity; ++, Moderate intensity; +++, Strong intensity.
