# Supplementary figures and images for: Defucosylated Monoclonal Antibody (H2Mab-139-mG2a-f) Exerted Antitumor Activities in Mouse Xenograft Models of Breast Cancers against Human Epidermal Growth Factor Receptor 2
Source: Curr Issues Mol Biol. 2023 Sep 23;45(10):7734–48. doi: 10.3390/cimb45100488 (PMC10605610; doi:10.3390/cimb45100488)

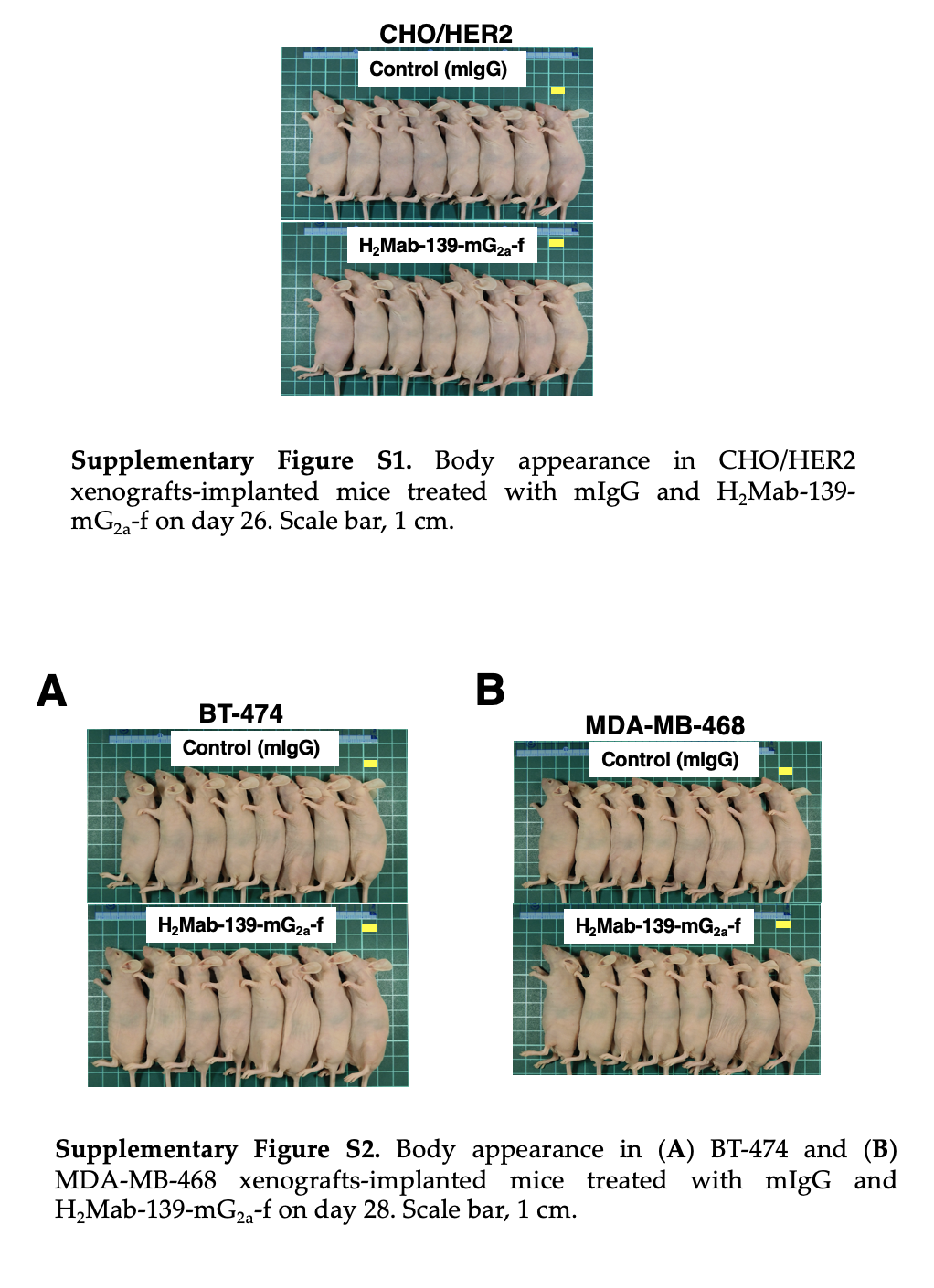

Supplement: Supplementary file 1 [file cimb-45-00488-s001.zip › Fig S1 & 2.png]
